# Supplementary material for: Availability, price and nutritional assessment of plant-based meat alternatives in hypermarkets and supermarkets in Petaling, the most populated district in Malaysia
Source: PLoS One. 2024 Dec 12;19(12):e0309507. doi: 10.1371/journal.pone.0309507 (PMC11637325; doi:10.1371/journal.pone.0309507)
Supplement: S1 Table — (DOCX) [file pone.0309507.s001.docx]

| **S1 Table.** PBMA Product Categories and Their Descriptions.   \| **Category** \| **Description** \| \| --- \| --- \| \| Burger/Patties \| Plant-Based Burger, Patties \| \| Coated Meat \| Plant-Based Breaded Items, Nuggets, Fingers,  Sticks, Tempura-Coated \| \| Luncheon Meat \| Plant-Based Luncheon Meat, Corned Meat \| \| Minced Meat \| Minced Plant-Based Meat \| \| Pastries \| Plant-Based Puffs, Spring Rolls, Popiah, Gyoza,  Dumplings \| \| Pieces/Chunks/Fillets/Strips \| Plant-Based Chunks, Strips, Fillets \| \| Prepacked Cooked Meals \| Plant-Based Pasta, Fried Rice, Ready-to-Eat Meals,  Products with Sauce \| \| Sausages \| Plant-Based Sausages, Hot Dog, Frankfurters \| \| Seafood Balls/Cakes/Meatballs \| Plant-Based Meatballs, Seafood Tofu, Fish Cakes \| |
| --- | --- | --- | --- | --- | --- | --- | --- | --- | --- | --- | --- | --- | --- | --- | --- | --- | --- | --- | --- | --- |
