# Supplementary material for: Availability, price and nutritional assessment of plant-based meat alternatives in hypermarkets and supermarkets in Petaling, the most populated district in Malaysia
Source: PLoS One. 2024 Dec 12;19(12):e0309507. doi: 10.1371/journal.pone.0309507 (PMC11637325; doi:10.1371/journal.pone.0309507)
Supplement: S2 Table — (DOCX) [file pone.0309507.s002.docx]

| **S2 Table.** Distribution of PBMAs Across Visited Supermarkets and Hypermarkets in Petaling District, Malaysia.   \| Store Categories \| Supermarket Name \| Location \| Coordinates \| Number of PBMAs Available \| \| --- \| --- \| --- \| --- \| --- \| \| Hypermarket \| AEON Big \| Puchong Utama \| 2.9914° N, 101.6144° E \| 40 \| \| Subang Jaya \| 3.0817° N, 101.5873° E \| 42 \| \| Jaya One \| 3.1171° N, 101.6351° E \| 35 \| \| Seksyen 23 Shah Alam \| 3.0444° N, 101.5260° E \| 45 \| \| Bukit Rimau \| 2.9995° N, 101.5284° E \| 37 \| \| Lotus’s \| Ara Damansara \| 3.119413° N, 101.576080° E \| 32 \| \| Mutiara Damansara \| 3.159586°N,101.613483°E \| 33 \| \| Paradigm Mall \| 1.5156° N, 103.6857° E \| 32 \| \| Shah Alam \| 3.072619° N, 101.538679° E \| 15 \| \| Setia Alam \| 3.106944°N,101.444444°E \| 14 \| \| Puchong \| 3.0348° N, 101.6159° E \| 30 \| \| Bukit Puchong \| 2.9824° N, 101.6175° E \| 18 \| \| HeroMarket \| Bandar Puteri Puchong \| 3.025586°N,101.618117°E \| 24 \| \| Kg. Bt. 14 \| 2.995818° N, 101.621360° E \| 19 \| \| Meranti Jaya \| 2.980259° N, 101.611582° E \| 11 \| \| Alam Megah \| 3.010802° N, 101.558021° E \| 20 \| \| Bandar Sri Damansara \| 3.198631° N, 101.618337° E \| 12 \| \| TTDI \| 3.1369°N,101.6306°E \| 18 \| \| Kelana Jaya \| 3.106496° N, 101.599574° E \| 15 \| \| Kota Kemuning \| 3.005848° N, 101.537564° E \| 22 \| \| Lapangan Terbang Lama Subang \| 3.144589° N, 101.538622° E \| 6 \| \| NSK Trade City \| One City \| 3.024444°N,101.579167°E \| 45 \| \| Star Avenue \| 3.154722°N,101.552500°E \| 36 \| \| Kota Damansara \| 3.159167°N,101.591944°E \| 36 \| \| Mydin \| Subang Jaya \| 3.059588° N, 101.596469° E \| 26 \| \| Giant \| USJ \| 3.0586°N,101.5947°E \| 28 \| \| BK5 \| 3.0507° N, 101.6456° E \| 38 \| \| Putra Heights \| 2.9897° N, 101.5723° E \| 10 \| \| Sri Manja \| 3.0744°N,101.6503°E \| 5 \| \| Kota Damansara \| 3.1561°N,101.5967°E \| 26 \| \| Shah Alam \| 3.0842°N,101.5494°E \| 20 \| \| Kemuning Utama \| 3.0186°N,101.5369°E \| 30 \| \| TF Value Mart \| USJ \| 3.032773° N, 101.588596° E \| 23 \| \| Sri Damansara \| 3.202358° N, 101.618696° E \| 21 \| \|  \|  \|  \| **Total** \| **864** \| \| Supermarket \| Giant Supermarket \| Kelana Jaya SS6 \| 3.1033°N,101.5978°E \| 2 \| \| AEON Supermarket \| Setia Alam Setia City Mall \| 3.1095° N, 101.4602° E \| 54 \| \| Shah Alam \| 3.0767°N,101.5477°E \| 44 \| \| IOI Mall Puchong \| 3.0457° N, 101.6182° E \| 32 \| \| OTK \| Puchong Prima \| 3.000000°N,101.595833°E \| 18 \| \| Redtick \| SetiaWalk Mall \| 3.0310° N, 101.6168° E \| 50 \| \| NSK Grocer \| Summit USJ \| 3.060000°N,101.593056°E \| 20 \| \| Amcorp Mall \| 3.105000°N,101.646667°E \| 14 \| \| 3 Damansara PJ \| 3.130278°N,101.626944°E \| 51 \| \| Taman Sri Muda \| 3.027038° N, 101.542640° E \| 61 \| \|  \|  \|  \| **Total** \| **346** \| \| Premium Supermarket \| Ben’s Independent Grocer \| Plaza Batai \| 3.1496°N, 101.6617°E \| 33 \| \| Publika \| 3.1720°N, 101.6644°E \| 44 \| \| IPC \| 3.1565°N, 101.6111°E \| 53 \| \| Jaya Grocer \| Sunway GEO \| 3.0652° N, 101.6099° E \| 31 \| \| Sunway Pyramid \| 3.0721° N, 101.6063° E \| 5 \| \| IOI Rio City \| 3.017756° N, 101.624381° E \| 30 \| \| Main Place Mall \| 3.0261°N,101.5811°E \| 38 \| \| Da Men \| 3.05867° N, 101.59331° E \| 66 \| \| NU Empire \| 3.0818° N, 101.5828° E \| 45 \| \| SS15 Courtyard \| 3.07722°N,101.58631°E \| 39 \| \| The Glades Putra Heights \| 3.0090° N, 101.5761° E \| 16 \| \| DC Mall \| 3.1455° N, 101.6625° E \| 37 \| \| Jaya 33 \| 3.1099° N, 101.6383° E \| 34 \| \| The Starling \| 3.1352° N, 101.6230° E \| 40 \| \| Glo Damansara \| 3.13389°N,101.62933°E \| 40 \| \| Pearl Point Shopping Mall \| 3.0847° N, 101.6733° E \| 58 \| \| Evolve Concept Mall \| 3.1101° N, 101.5865° E \| 40 \| \| 1 Utama \| 3.1481° N, 101.6164° E \| 45 \| \| Perdana Shopping Centre \| 3.1679° N, 101.6060° E \| 26 \| \| Mutiara Tropicana \| 3.1288° N, 101.6001° E \| 32 \| \| Centrepoint \| 3.1375° N, 101.6098° E \| 19 \| \| Plaza Shah Alam \| 3.0739°N,101.5170°E \| 36 \| \| Quayside Mall \| 2.9567°N,101.5528°E \| 45 \| \| Emporis Kota Damansara \| 3.1571° N, 101.5715° E \| 31 \| \| Ardence Labs \| 3.099023° N, 101.477737° E \| 35 \| \| The Food Merchant \| Pavillion Bukit Jalil \| 3.0502° N, 101.6710° E \| 65 \| \| Prelude Pavillion Damansara \| 3.146389°N,101.663056°E \| 35 \| \| Village Grocer \| Gamuda Walk Mall \| 3.0006° N, 101.5335° E \| 32 \| \| Subang Parade \| 3.082222°N,101.585556°E \| 43 \| \| Megah Rise Mall \| 3.115108° N, 101.612131° E \| 66 \| \| Atria Mall \| 3.127242° N, 101.616615° E \| 3 \| \| Citta Mall \| 3.149730°N,101.593656°E \| 64 \| \| Tropicana Avenue \| 3.129065°N,101.600120°E \| 20 \| \| Tropicana Gardens Mall \| 3.1492° N, 101.5931° E \| 68 \| \| Sunway Giza Mall \| 3.150833°N,101.591389°E \| 86 \| \| Central i-City \| 3.0624° N, 101.4817° E \| 59 \| \| Sunsuria Forum \| 3.1053° N, 101.4671° E \| 67 \| \|  \|  \|  \| **Total** \| **1526** \| |
| --- | --- | --- | --- | --- | --- | --- | --- | --- | --- | --- | --- | --- | --- | --- | --- | --- | --- | --- | --- | --- | --- | --- | --- | --- | --- | --- | --- | --- | --- | --- | --- | --- | --- | --- | --- | --- | --- | --- | --- | --- | --- | --- | --- | --- | --- | --- | --- | --- | --- | --- | --- | --- | --- | --- | --- | --- | --- | --- | --- | --- | --- | --- | --- | --- | --- | --- | --- | --- | --- | --- | --- | --- | --- | --- | --- | --- | --- | --- | --- | --- | --- | --- | --- | --- | --- | --- | --- | --- | --- | --- | --- | --- | --- | --- | --- | --- | --- | --- | --- | --- | --- | --- | --- | --- | --- | --- | --- | --- | --- | --- | --- | --- | --- | --- | --- | --- | --- | --- | --- | --- | --- | --- | --- | --- | --- | --- | --- | --- | --- | --- | --- | --- | --- | --- | --- | --- | --- | --- | --- | --- | --- | --- | --- | --- | --- | --- | --- | --- | --- | --- | --- | --- | --- | --- | --- | --- | --- | --- | --- | --- | --- | --- | --- | --- | --- | --- | --- | --- | --- | --- | --- | --- | --- | --- | --- | --- | --- | --- | --- | --- | --- | --- | --- | --- | --- | --- | --- | --- | --- | --- | --- | --- | --- | --- | --- | --- | --- | --- | --- | --- | --- | --- | --- | --- | --- | --- | --- | --- | --- | --- | --- | --- | --- | --- | --- | --- | --- | --- | --- | --- | --- | --- | --- | --- | --- | --- | --- | --- | --- | --- | --- | --- | --- | --- | --- | --- | --- | --- | --- | --- | --- | --- | --- | --- | --- | --- | --- | --- | --- | --- | --- | --- | --- | --- | --- | --- | --- | --- | --- | --- | --- | --- | --- | --- | --- | --- | --- | --- | --- | --- | --- | --- | --- | --- | --- | --- | --- | --- | --- | --- | --- | --- |
