# Supplementary material for: Availability, price and nutritional assessment of plant-based meat alternatives in hypermarkets and supermarkets in Petaling, the most populated district in Malaysia
Source: PLoS One. 2024 Dec 12;19(12):e0309507. doi: 10.1371/journal.pone.0309507 (PMC11637325; doi:10.1371/journal.pone.0309507)
Supplement: S3 Table — (DOCX) [file pone.0309507.s003.docx]

| **S3 Table.** Mean Energy Density and Nutritional Contents (per 100g) ± Standard Deviations (Range) of Plant-Based vs. Meat-Based Product Categories.   \| Meat/  Plant-Based \| Product Categories (Sample Size) \| Energy (kcal) \| *p* \| Protein (g) \| *p* \| Total Fat (g) \| *p* \| Saturated Fat (g) \| *p* \| Carbohydrates (g) \| *p* \| Sodium (mg) \| *p* \| \| --- \| --- \| --- \| --- \| --- \| --- \| --- \| --- \| --- \| --- \| --- \| --- \| --- \| --- \| \| MB \| Burger/Patties (n=3) \| 287.67±104.17  (206–405) \| 0.2927 \| 18.56±3.69  (15.2-22.5) \| 0.1174 \| 22.29±15.12  (11.5-39.6) \| 0.3090 \| 7.70±4.56  (4.1-12.8) \| 0.4157 \| 4.63±3.11  (1.4-7.6) \| 0.0182* \| 460±242.02  (243-721) \| 0.7843 \| \| PB \| Burger/Patties (n=20) \| 202.82±40.54 (127-265) \| 13.45±4.93 (2.3-21) \| 10.50±4.32 (5-18) \| 4.97±4.9  (0.4-16.8) \| 12.36±8.34  (2-30) \| 504.23±143.41  (309.7-881) \| \| MB \| Coated Meat  (n=4) \| 255±85.08  (178-366) \| 0.9844 \| 12±1.92  (10.1-13.9) \| 0.6988 \| 15.42±7.44  (7.6-24.5) \| 0.6359 \| 5.18±3.88  (3.1-11) \| 0.9243 \| 16.40±6.40  (12-25.9) \| 0.5764 \| 581.50±634.01  (128-1515) \| 0.9034 \| \| PB \| Coated Meat (n=55) \| 254.07±82.76 (140-613) \| 12.50±5.82  (2-24) \| 13.42±7.22 (0.1-34) \| 5.39±4.11  (0-16) \| 18.49±9.47  (0-54.8) \| 539.46±297.34  (80-1543.9) \| \| MB \| Luncheon Meat  (n=6) \| 169.51±86.76  (80.1-313) \| 0.2168 \| 17.17±4.72  (9.3-23.6) \| 0.0226* \| 10.75±10.26  (1.1-28.8) \| 0.3744 \| 4.60±3.95  (0.5-11) \| 0.9582 \| 0.75±1.69  (0-4.2) \| 0.1081 \| 1259.33±313.77  (835-1670) \| 0.0032  ** \| \| PB \| Luncheon Meat (n=3) \| 220.67±18.01 (203-239) \| 10.87±1.59 (9.9-12.7) \| 14.87±1.18 (13.5-15.6) \| 4.40±5.54  (1.2-10.8) \| 9.10±5.37  (2.9-12.2) \| 615.33±118.32  (485-716) \| \| MB \| Minced Meat  (n=3) \| 194.42±61.81  (143-263) \| 0.1637 \| 18.14±1.72  (16.9-20.1) \| 0.0272* \| 13.40±6.97  (8.1-21.3) \| 0.1566 \| 4.69±2.87  (2.3-7.9) \| 0.1773 \| 0.01±0.02  (0-0) \| 0.1214 \| 60±4  (56-64) \| 0.0631 \| \| PB \| Minced Meat  (n=4) \| 116.50±59.90 (66-203) \| 13.53±0.90 (12.8-14.8) \| 4.65 ±6.06 (0.9-13.7) \| 1.40±2.08  (0-4.5) \| 3.73±3.46  (1.6-8.9) \| 250.03±131.67  (55.1-340) \| \| MB \| Pastries  (n=15) \| 288.46±110.04  (134.1-506) \| 0.0080  ** \| 7.32±2.59  (1.7-11.4) \| 0.5731 \| 17.01±7.45  (8-30.9) \| <0.001  ** \| 8±5.56  (0-21.4) \| 0.0085  ** \| 26.46±11.84  (8.9-46.1) \| 0.1851 \| 441.51±163.96  (281-916) \| 0.2308 \| \| PB \| Pastries  (n=21) \| 197.58 ±51.50 (90-313.1) \| 7.95±4.06 (3.9-20.8) \| 8.55±5.42 (0.1-21.3) \| 3.29±3.20  (0-9.6) \| 21.74±7.35  (4.7-33.9) \| 380.93±115.31  (170-638) \| \| MB \| Pieces/Chunks/  Fillets/Strips (n=53) \| 148.51±86.43  (33-476) \| 0.0016  ** \| 18.30±4.36  (2.9-26.3) \| <0.001  ** \| 6.69±8.30  (0-34.9) \| 0.1496 \| 2.65±3.80  (0-18) \| 0.0311* \| 1.70±4.42  (0-18.9) \| <0.001  ** \| 118.95±116.81  (6-658) \| <0.001  ** \| \| PB \| Pieces/Chunks/  Fillets/Strips (n=60) \| 203.52±94.43 (23.4-557) \| 13.32±5.66 (0.4-27.4) \| 8.93±8.06  (0.1-38.1) \| 4.34±4.41  (0-17.8) \| 15.47±7.69  (1.7-45.5) \| 582.36±478.03  (0.6-3662) \| \| MB \| Prepacked Cooked  Meals (n=10) \| 196.90±70.84  (93.9-323) \| 0.9077 \| 13.54±4.50  (7.9-22.2) \| 0.0014  ** \| 12.31±6.33  (1.6-20.4) \| 0.2563 \| 4.35±3.17  (0-10.9) \| 0.9854 \| 8.19±5.16  (1.4-19.2) \| <0.001  ** \| 797.70±451.42  (226-1421) \| 0.4760 \| \| PB \| Prepacked Cooked  Meals (n=25) \| 200.44±100.74 (82-585.1) \| 7.16±4.02 (2.3-18.5) \| 9.02±10.01 (0.2-51.8) \| 4.32±5.01  (0-24.1) \| 22.38±10.91  (8.7-47.7) \| 646.92±760.96  (0-2972) \| \| MB \| Sausages  (n=9) \| 269.67±68.01  (151-360) \| 0.0033  ** \| 16.40±5.12  (11.7-28.6) \| 0.5393 \| 19.13±8.38  (5.9-28.5) \| 0.0113* \| 7.37±3.78  (2.5-12.5) \| 0.0098  ** \| 7.58±13.08  (0.7-40.9) \| 0.6424 \| 1049.56±311  (624-1475) \| <0.001  ** \| \| PB \| Sausages  (n=8) \| 157.76±64.18 (14-205) \| 14.95±4.41 (7.7-22.4) \| 9.14±5.62 (3.2-19.7) \| 2.88±2.27  (0-6) \| 9.75±3.53  (4-13.8) \| 522.17±200.37  (61.3-670) \| \| MB \| Seafood Balls/  Cakes/Meatballs (n=6) \| 152.33±55.02  (83-207) \| 0.3865 \| 11.58±1.98  (8.3-13.5) \| 0.1693 \| 11.58±1.98  (8.3-13.5) \| 0.6621 \| 2.77±2.39  (0-6.2) \| 0.1884 \| 7.27±4.66  (0.2-12.1) \| 0.0231* \| 654.17±170.70  (386-822) \| 0.0364* \| \| PB \| Seafood Balls/  Cakes/Meatballs (n=20) \| 178.79±85.70 (0-313.1) \| 9.41±5.81  (1-20.8) \| 9.75±7.77  (0-23.2) \| 4.63±4.15  (0-13.3) \| 13.59±6.79  (7.2-38) \| 457.58±147.73  (260-750) \|   **p-value significant at < 0.05*  **** *p-value significant at < 0.01* |
| --- | --- | --- | --- | --- | --- | --- | --- | --- | --- | --- | --- | --- | --- | --- | --- | --- | --- | --- | --- | --- | --- | --- | --- | --- | --- | --- | --- | --- | --- | --- | --- | --- | --- | --- | --- | --- | --- | --- | --- | --- | --- | --- | --- | --- | --- | --- | --- | --- | --- | --- | --- | --- | --- | --- | --- | --- | --- | --- | --- | --- | --- | --- | --- | --- | --- | --- | --- | --- | --- | --- | --- | --- | --- | --- | --- | --- | --- | --- | --- | --- | --- | --- | --- | --- | --- | --- | --- | --- | --- | --- | --- | --- | --- | --- | --- | --- | --- | --- | --- | --- | --- | --- | --- | --- | --- | --- | --- | --- | --- | --- | --- | --- | --- | --- | --- | --- | --- | --- | --- | --- | --- | --- | --- | --- | --- | --- | --- | --- | --- | --- | --- | --- | --- | --- | --- | --- | --- | --- | --- | --- | --- | --- | --- | --- | --- | --- | --- | --- | --- | --- | --- | --- | --- | --- | --- | --- | --- | --- | --- | --- | --- | --- | --- | --- | --- | --- | --- | --- | --- | --- | --- | --- | --- | --- | --- | --- | --- | --- | --- | --- | --- | --- | --- | --- | --- | --- | --- | --- | --- | --- | --- | --- | --- | --- | --- | --- | --- | --- | --- | --- | --- | --- | --- | --- | --- | --- | --- | --- | --- | --- | --- | --- |
