# Supplementary material for: Availability, price and nutritional assessment of plant-based meat alternatives in hypermarkets and supermarkets in Petaling, the most populated district in Malaysia
Source: PLoS One. 2024 Dec 12;19(12):e0309507. doi: 10.1371/journal.pone.0309507 (PMC11637325; doi:10.1371/journal.pone.0309507)
Supplement: S4 Table — (DOCX) [file pone.0309507.s004.docx]

**S4 Table.** Nutri-Score Classification across PBMA Product Categories.

| Product Categories (Sample Size) | Mean Nutri-Score ± SD | Frequency of Assigned Nutri-Score Colors | | | | |
| --- | --- | --- | --- | --- | --- | --- |
|  |  | **Red** | **Orange** | **Yellow** | **Light Green** | **Dark Green** |
| Burger/Patties  (*n* = 15) | 3.80±6.20 | 0 | 3 | 6 | 1 | 5 |
| Coated Meat  (*n* = 48) | 5.85±8.21 | 4 | 10 | 17 | 2 | 15 |
| Luncheon Meat  (*n* = 3) | 7.67±3.79 | 0 | 1 | 2 | 0 | 0 |
| Minced Meat  (*n* = 4) | -9.25±4.27 | 0 | 0 | 0 | 0 | 4 |
| Pastries  (*n* = 13) | 2.46±5.99 | 0 | 1 | 5 | 0 | 7 |
| Pieces/Chunks/Fillets/Strips (*n* = 45) | 4.96±7.96 | 1 | 12 | 16 | 0 | 16 |
| Prepacked Cooked Meals (n = 17) | 7.88±8.20 | 1 | 6 | 4 | 2 | 4 |
| Sausages  (*n* = 8) | 0.25±7.69 | 0 | 0 | 3 | 0 | 5 |
| Seafood Balls/Cakes/Meatballs (*n* = 17) | 3.00±6.54 | 0 | 3 | 6 | 2 | 6 |
