# Supplementary material for: Availability, price and nutritional assessment of plant-based meat alternatives in hypermarkets and supermarkets in Petaling, the most populated district in Malaysia
Source: PLoS One. 2024 Dec 12;19(12):e0309507. doi: 10.1371/journal.pone.0309507 (PMC11637325; doi:10.1371/journal.pone.0309507)
Supplement: S5 Table — (DOCX) [file pone.0309507.s005.docx]

**S5 Table**. Mean Energy Density and Nutritional Contents (per 100g) of Malaysian PBMAs vs. European PBMAs and Meat Equivalents.

| Country/  Region | Product Categories | Energy (kcal) | Protein (g) | Total Fat (g) | Saturated Fat (g) | Carbohydrates (g) | Sodium (mg) |
| --- | --- | --- | --- | --- | --- | --- | --- |
| Malaysia | PB Burger/Patties | 202.82 | 13.45 | 10.50 | 4.97 | 12.36 | 504.23 |
| Europe | PB Burger/Patties | 201.32 | 12.88 | 10.84 | 1.74 | 10.19 | 483.46 |
|  | MB Burger/Patties | 219.72 | 17.22 | 14.98 | 6.30 | 2.61 | 441.73 |
| Malaysia | PB Coated Meat | 254.07 | 12.50 | 13.42 | 5.39 | 18.49 | 539.46 |
| Europe | PB Coated Meat | 225.45 | 13.60 | 10.50 | 1.20 | 16.00 | 472.44 |
|  | MB Coated Meat | 262.45 | 14.65 | 15.50 | 2.90 | 2.50 | 393.70 |
| Malaysia | PB Minced Meat | 116.50 | 13.53 | 4.65 | 1.40 | 3.73 | 250.03 |
| Europe | PB Minced Meat | 178.48 | 17.60 | 8.40 | 1.83 | 5.12 | 370.08 |
|  | MB Minced Meat | 191.65 | 19.99 | 12.18 | 5.33 | 0.48 | 108.27 |
| Malaysia | PB Pieces/Chunks/  Fillets/Strips | 203.52 | 13.32 | 10.53 | 4.34 | 15.47 | 582.36 |
| Europe | PB Pieces/Chunks/  Fillets/Strips | 170.93 | 16.60 | 8.30 | 1.10 | 5.17 | 643.70 |
|  | MB Pieces/Chunks/  Fillets/Strips | 111.68 | 16.90 | 4.59 | 1.65 | 0.51 | 107.87 |
| Malaysia | PB Sausages | 157.76 | 14.95 | 9.14 | 2.88 | 9.75 | 522.17 |
| Europe | PB Sausages | 201.68 | 14.25 | 12.44 | 1.87 | 5.44 | 676.18 |
|  | MB Sausages | 254.63 | 12.74 | 19.71 | 7.42 | 5.32 | 694.88 |
| Malaysia | PB Seafood Balls/Cakes/Meatballs | 178.79 | 9.41 | 9.75 | 4.63 | 13.59 | 457.58 |
| Europe | PB Seafood Balls/Cakes/Meatballs | 195.10 | 13.45 | 10.06 | 1.18 | 10.95 | 521.65 |
|  | MB Seafood Balls/Cakes/Meatballs | 198.90 | 16.15 | 12.60 | 4.98 | 5.13 | 561.02 |

European countries include United Kingdom, Italy, Sweden, Germany, and Norway. All European values were derived from the studies of Alessandrini et al., 2021; Bryngelsson et al., 2022; Cutroneo et al., 2022; Pointke & Pawelzik, 2022; Tonheim et al., 2022.
