# Supplementary material for: Availability, price and nutritional assessment of plant-based meat alternatives in hypermarkets and supermarkets in Petaling, the most populated district in Malaysia
Source: PLoS One. 2024 Dec 12;19(12):e0309507. doi: 10.1371/journal.pone.0309507 (PMC11637325; doi:10.1371/journal.pone.0309507)
Supplement: Attachment — Submitted filename: Review report on PBMA_Plose one.docx [file pone.0309507.s006.docx]

It is pleasure to me as a reviewer to review this paper. The paper is well structured and written in scientifically standards but I recommended little comments to revise in the paper.

**Introduction**

1. Page 4, line #103, you have stated that there are many studies explaining the nutritional profile and healthiness of PBMAs in other areas which lack references supporting your evidence. So please cite references there that support this argument.

**Methods**

1. Under methodology section, line #113- is scientifically sound to frame the sub-title as Study site, sample selection and collection.

**Results**

1. Page 9, lines #201-#206, the lines are written in jungle sentences; please improve the sentences.
2. Page 9, line #208, discusses the three countries in which those PBMAs products originated.
3. Page 9, lines #220- #221, it is better if you write the prices for the mentioned food products.
4. Page 10, lines #226- #230, look over the sentences again to improve the grammar and language.
5. I suggest to put findings in amounts rather than writing in text throughout the result section.
6. Page 11, line #252, indicates the measurement unit for the mean finding of 2.96 Nutri-Score.

**Discussion**

1. Can you discuss in the discussion section the drawback of processed foods, which results in unhealthy foods because you have only explained that consumption of such processed foods is increasing across the globe? Your argument delves more into processed foods from premium supermarkets, hypermarkets, and supermarkets, which can serve as healthy foods for most consumers, which may ignore the narrative of the effect of processed foods on health.
2. For example, what about your implication for jungle foods like Burger, which is a category of unhealthy food?
3. Overall, please remove the results presentation from the discussion section and try to justify the possible reasons and consequences, and discuss if there are similar or different findings with previous works.

**Figures**

1. The figures are not clearly visible; for example, the texts, numbers, and proportions are presented in a font size that is too small to effectively describe the results. Therefore, I suggest improving the figures by adhering to standard formatting figures.
