# Supplementary material for: Availability, price and nutritional assessment of plant-based meat alternatives in hypermarkets and supermarkets in Petaling, the most populated district in Malaysia
Source: PLoS One. 2024 Dec 12;19(12):e0309507. doi: 10.1371/journal.pone.0309507 (PMC11637325; doi:10.1371/journal.pone.0309507)
Supplement: Attachment — Submitted filename: PONE-D-24-34533 - Reviewre comments.docx [file pone.0309507.s008.docx]

Thank you for the opportunity to review Manuscript Number *PONE-D-24-34533* for the Journal of PLOS ONE. This scientific research study titled "Availability, Price, and Nutritional Assessment of Plant-Based Meat Alternatives in Hypermarkets and Supermarkets in Petaling, the Most Populated District in Malaysia" presents intriguing findings and will significantly enhance the understanding of plant-based food markets in the field of science.

As **Reviewer**, I would like to highlight several key points regarding PLOS ONE criteria:

Why The manuscript's preprint is released in August 2024 on ResearchGate, medRxiv and Sciety?

DOI:10.1101/2024.08.15.24312042
<https://www.researchgate.net/publication/383189120_Availability_Price_and_Nutritional_Assessment_of_Plant-Based_Meat_Alternatives_in_Hypermarkets_and_Supermarkets_in_Petaling_the_Most_Populated_District_in_Malaysia> <https://www.medrxiv.org/content/10.1101/2024.08.15.24312042v1> <https://labs.sciety.org/articles/by?article_doi=10.1101/2024.08.15.24312042>

| Page | Line |  |  |
| --- | --- | --- | --- |
| - | - | 1. The study presents the results of original research.  2. Results reported have not been published elsewhere. | Except the manuscript's preprint is released on ResearchGate, medRxiv and Sciety, the study presents original research findings that are well-structured, clearly analysed, and contribute valuable insights to the field. |
| - | - | 3. Experiments, statistics, and other analyses are performed to a high technical standard and are described in sufficient detail. | For the statistics:  The study employs a high standard of statistical analysis, utilizing appropriate methods such as the Shapiro-Wilk test for normality and defining clear criteria for significance. Additionally, the details provided on data compilation and the use of the R statistical environment enhance the transparency and reproducibility of the analysis.  For the Experiments:   - The experimental tasks were executed with a commendable technical standard, particularly in the systematic approach to sample selection and product categorization. Furthermore, the detailed descriptions of the methods used for availability assessment, price analysis, and nutrient profiling provide clarity and allow for reproducibility.   e.g. Availability Assessment (definitions, categories)  The Malaysian-German Chamber of Commerce and Industry (MGCC).   - The clear identification of exclusion criteria, including the specification that six items lacking price or packaging size data were excluded, strengthens the study's rigor and transparency. - The decision to source saturated fat information from Singapore's Energy & Nutrient Composition Database due to its unavailability in the Malaysian Food Composition Database demonstrates a thorough and reasonable approach to ensure comprehensive nutrient profiling. - The use of the Nutri-Score Nutritional Rating System for assessing plant-based meat alternatives, along with clear definitions of the grading criteria and the presentation of mean scores and frequencies by product category, enhances the clarity and comprehensiveness of the nutrient profiling methodology. |
| - | - | 4. Conclusions are presented in an appropriate fashion and are supported by the data. | The conclusions were presented clearly and are well-supported by the data, effectively highlighting the potential benefits and limitations of plant-based meat alternatives while recommending further research to deepen understanding of their micronutrient profiles. |
| - | - | 5. The article is presented in an intelligible fashion and is written in standard English. | The article is articulately presented and is written in clear, standard English, making it accessible to a broad audience. |
| - | - | 6. The research meets all applicable standards for the ethics of experimentation and research integrity. | The ethical statement effectively clarifies that the research complies with appropriate ethical standards, affirming that it does not involve human or animal subjects or sensitive data. |
| - | - | 7. The article adheres to appropriate reporting guidelines and community standards for data availability. | The article satisfactorily adheres to reporting guidelines regarding data availability, specifying that data can be accessed upon request, while also providing detailed supporting information that enhances the transparency and reproducibility of the research findings. |
| 26-29 | - | Figures | The figures in the manuscript are presented in poor resolution, which hinders the clarity and interpretability of the data being displayed. |
| 30 | - | Tables | Could the author clarify the rationale for placing the tables in the supporting information instead of including them directly in the manuscript? |
| 2 | 26-45 | Abstract | The abbreviation "PBMAs" for Plant-Based Meat Alternatives should be clearly defined in the abstract for clarity, ensuring readers unfamiliar with the term can fully understand the context. |
| 7-8 | 150-193 | Availability Assessment  Price Analysis  Energy and Nutrient Content Comparison  Nutrient Profiling | The tasks listed, Availability Assessment, Price Analysis, Energy and Nutrient Content Comparison, and Nutrient Profiling, should be included in the Methods section rather than under Statistical Analysis to improve the organization and flow of the manuscript for the reader. |
| 7 | 153 | Availability Assessment | Please ensure that there is a single space between the word and reference in the manuscript.  e.g. in the sentence "… provided by the Malaysian-German Chamber of Commerce and Industry (MGCC)[25], these …" |
| 16-17 | 397-413 | Conclusions | Why are some key findings highlighted in the abstract but not reiterated or sufficiently addressed in the conclusion?  In abstract  These findings suggest that PBMAs offer a viable alternative for consumers in Malaysia seeking healthier and more sustainable food options, particularly for those aiming to reduce their intake of fat and sodium. However, PBMAs may not be recommended for individuals seeking a higher protein and lower carbohydrate diet. Further research is recommended to explore micronutrient profiles to enhance dietary decision-making.  In conclusion  Considering these findings, PBMAs present themselves as a viable choice for consumers in Malaysia in terms of availability, price, and nutritional composition (except carbohydrate). However, further research should include direct comparisons with local meat equivalents, encompassing both processed and unprocessed varieties, to better understand the complete nutritional benefits and limitations of PBMAs. Additionally, a thorough assessment of micronutrient content is essential to fully assess the health implications of these products, ensuring that consumers are well-informed about their dietary choices. |
