# Supplementary material for: Availability, price and nutritional assessment of plant-based meat alternatives in hypermarkets and supermarkets in Petaling, the most populated district in Malaysia
Source: PLoS One. 2024 Dec 12;19(12):e0309507. doi: 10.1371/journal.pone.0309507 (PMC11637325; doi:10.1371/journal.pone.0309507)
Supplement: Attachment — Submitted filename: PLOSONE PBMA Response to Reviewers.docx [file pone.0309507.s009.docx]

**PONE-D-24-34533**

**Availability, Price and Nutritional Assessment of Plant-Based Meat Alternatives in Hypermarkets and Supermarkets in Petaling, the Most Populated District in Malaysia**

**Katty Jia Qi Lou, Nadia Nantheni Rajaram, Yee-How Say**

**PLOS ONE**

Dear Academic Editor,

We thank you and the reviewers for the constructive comments and we have addressed them point-by-point and amended the manuscript, as follows:

| **Comments** | **Response to Reviewers** |
| --- | --- |
| **Reviewer #1** |  |
| 1. The study mentions that PBMAs generally have lower protein content compared to meat equivalents. What specific strategies do you suggest for improving the protein content of PBMAs in the Malaysian market? | Recommendation is provided as follows in Discussion:  “To significantly increase the protein content of Malaysian PBMAs, food manufacturers should diversify and intensify protein-rich ingredients like soy protein isolate, pea protein, lentils, chickpeas, beans, textured vegetable protein, wheat gluten, hemp seeds, pumpkin seeds, almonds, and mycoprotein [42] into their formulation.” |
| 2. Given the findings on the nutritional quality of PBMAs, how do you plan to disseminate this information to consumers to help them make informed dietary choices? | Recommendation is provided in the final sentence of Conclusions:  “To improve awareness of the nutritional quality of PBMAs in Malaysia, a voluntary front-of-pack labelling system should be encouraged for all PBMA products.” |
| 3. Have you considered conducting follow-up studies to assess changes in the availability and pricing of PBMAs over time, especially as consumer demand increases? | We have added this as one of the limitations of study:  “Lastly, the cross-sectional nature of this study makes us unable to evaluate the availability and pricing of PBMAs over time, especially as consumer demand increases. Hence, follow-up studies – like 6 months or 1 year later - are needed.” |
| 4. You noted the need for further research on micronutrient profiles. What specific micronutrients do you believe are most critical to assess in future studies, and why? | Conclusions rewritten as follows:  “Additionally, a thorough assessment of micronutrient contents, especially zinc and iron, are essential to fully assess the health implications of these products, ensuring that consumers are well-informed about their dietary choices.” |
| 5. You mentioned limitations regarding the use of the Singapore database for meat equivalents. How might this impact the validity of your comparisons, and what steps could be taken in future research to address this issue? | Usage of the Singapore database for comparison is valid due to its geographical proximity, cultural similarity, and the fact that it imports most of its food products from Malaysia.  The section “Energy and Nutrient Content Comparison” in Methods has been rewritten.  The limitation is mostly based on raw or uncooked data in the Singapore database, whereas PBMAs surveyed in this study were mainly processed or cooked. Step to address this issue: A direct comparison could be performed in the future by surveying equivalent PBMA product items that are meat-based. |
| 6. The discussion touches on the health implications of ultra-processed foods. How do you propose to evaluate the degree of processing in PBMAs in future studies, and what impact do you think this has on consumer health? | Some of the critiques of evaluating the degree of processing of PBMAs solely using the NOVA system and automatically equating PBMAs as "ultra-processed" with poor nutritional quality have been added in Discussion. |
| 7. Considering Malaysia's diverse culinary landscape, how do you think cultural preferences might influence the acceptance and consumption of PBMAs? | Study on how cultural preferences might influence the acceptance and consumption of PBMAs in Malaysia is not within the scope of this study and has already been covered by these previous publications in Malaysia:   - Abu Bakar AR, Mahzan NA, Tarmazi SAA, Tumin SA. Plant-base Meat as Future Alternative: A Study of Malaysian Consumers Purchase Intention. MJSSH. 2023;8: e002397. doi:10.47405/mjssh.v8i7.2397 - Manickam HV. Purchase Intention Towards Plant-Based Food in Malaysia. UTAR Institutional Repository; 2022. Available: <http://eprints.utar.edu.my/4846/1/8._Hephzibah_Victor_1901444.pdf> - Tan XY, Cheng S-H. Understanding Malaysian consumers perception and acceptability of plant-based alternatives to chicken: Do texture, taste, and environmental impact matter? Food and Humanity. 2024;2: 100304. doi:10.1016/j.foohum.2024.100304 |
| **Reviewer #2** |  |
| **Introduction**   1. Page 4, line #103, you have stated that there are many studies explaining the nutritional profile and healthiness of PBMAs in other areas which lack references supporting your evidence. So please cite references there that support this argument. | References [1,2,9,11,13] added. |
| **Methods**   1. Under methodology section, line #113- is scientifically sound to frame the sub-title as Study site, sample selection and collection. | Subtitle renamed to **Study site, Sample Selection and Collection.** |
| **Results**   1. Page 9, lines #201-#206, the lines are written in jungle sentences; please improve the sentences. 2. Page 9, line #208, discusses the three countries in which those PBMAs products originated. 3. Page 9, lines #220- #221, it is better if you write the prices for the mentioned food products. 4. Page 10, lines #226- #230, look over the sentences again to improve the grammar and language. 5. I suggest to put findings in amounts rather than writing in text throughout the result section. 6. Page 11, line #252, indicates the measurement unit for the mean finding of 2.96 Nutri-Score. | 1. Lines #201-#206: Sentences rewritten as follows: Premium supermarkets offered the highest variety of PBMAs across all product categories, with the mean of 41.2 products per store (Figure 1B). This mean was significantly higher than hypermarkets (Mean: 25.4; *p* < 0.001), but not supermarkets (Mean: 34.6; *p* = 0.47). 2. Sentence rephrased to “Most product categories feature PBMA items originating from two or more additional countries besides Malaysia” for better clarity. 3. Median prices included in parenthesis after the mentioned food products. 4. Sentence rewritten for better clarity. 5. All the findings were amended to amounts (numbers) rather than writing in text. 6. Nutri-Score are in arbitrary units and therefore no unit is available for them. |
| **Discussion**   1. Can you discuss in the discussion section the drawback of processed foods, which results in unhealthy foods because you have only explained that consumption of such processed foods is increasing across the globe? Your argument delves more into processed foods from premium supermarkets, hypermarkets, and supermarkets, which can serve as healthy foods for most consumers, which may ignore the narrative of the effect of processed foods on health. 2. For example, what about your implication for jungle foods like Burger, which is a category of unhealthy food? 3. Overall, please remove the results presentation from the discussion section and try to justify the possible reasons and consequences, and discuss if there are similar or different findings with previous works. | 1. Critics argue that it is important not to assume that “ultra-processed” equals poor nutritional quality, since quality does not depend solely on the intensity or complexity of processing but on the final composition of the food itself. Additional sentences were added to the Nutri-Score paragraph in Discussion. 2. Explanation is the same as above. 3. Results presented in Discussion were the summary of the findings, with none of the numerical figures repeated. We had already justified the possible reasons and consequences, and similarity/differences from previous findings in Discussion. |
| **Figures**   1. The figures are not clearly visible; for example, the texts, numbers, and proportions are presented in a font size that is too small to effectively describe the results. Therefore, I suggest improving the figures by adhering to standard formatting figures. | Figures were unfortunately presented at a lower resolution in the pdf format of the file for reviewers. The original figures were all presented in high resolution and according to the standard formatting. We would appreciate if the reviewers could download the high-resolution figures from the “Click here to access/download” link located at the top right corner of the figures. Thank you. |
| **Reviewer #3:** |  |
| Suggestions: Enhance the description of the rationale behind the Nutri-Score in the methodological section for better clarity. | Rationale for Nutri-Score provided:  This scoring system was selected as it is a widely used front-of-pack labelling system that provides user-friendly information on the nutritional quality of food and beverages. This food-labelling system has been applied voluntarily in food products sold in European Union countries such as Sweden, Italy, and Germany; hence, nutrient profiling of PBMAs in these countries also utilised Nutri-Score [21–23]. |
| Doubts: Out of the total of 2,736 PBMAs, why were only 251 subject to the Energy and Nutrient Content Comparison and Nutri-Score Evaluation of Nutritional Quality analysis? Specifying the inclusion and exclusion criteria could strengthen the credibility of the analysis. | A total of 2736 PBMA product items were available from these 81 surveyed locations, with 251 being unique products manufactured by 42 brands.  The first paragraph of “Availability of PBMAs” in Results has been rewritten. |
| **Reviewer #4** |  |
| Why The manuscript's preprint is released in August 2024 on ResearchGate, medRxiv and Sciety? | We published in manuscript as preprint in <https://www.medrxiv.org/content/10.1101/2024.08.15.24312042v1>. This preprint has been picked up by other research paper repositories in the internet. |
| **Figures**  The figures in the manuscript are presented in poor resolution, which hinders the clarity and interpretability of the data being displayed. | Figures were unfortunately presented at a lower resolution in the pdf format of the file for reviewers. The original figures were all presented in high resolution and according to the standard formatting. We would appreciate if the reviewers could download the high-resolution figures from the “Click here to access/download” link located at the top right corner of the figures. Thank you. |
| **Tables**  Could the author clarify the rationale for placing the tables in the supporting information instead of including them directly in the manuscript? | Including the tables directly in the manuscript instead of supporting information would make the manuscript too lengthy. Some of the information in the tables is also repetitive of the figures. |
| **Abstract**  The abbreviation "PBMAs" for Plant-Based Meat Alternatives should be clearly defined in the abstract for clarity, ensuring readers unfamiliar with the term can fully understand the context. | The abbreviation for PBMAs has been now defined in Abstract. |
| The tasks listed, Availability Assessment, Price Analysis, Energy and Nutrient Content Comparison, and Nutrient Profiling, should be included in the Methods section rather than under Statistical Analysis to improve the organization and flow of the manuscript for the reader. | Availability Assessment, Price Analysis, Energy and Nutrient Content Comparison, and Nutrient Profiling moved to main sections in Methods, rather than as subsections under Statistical Analysis. |
| Availability Assessment  Please ensure that there is a single space between the word and reference in the manuscript.  e.g. in the sentence "… provided by the Malaysian-German Chamber of Commerce and Industry (MGCC)[25], these …" | Space added between MGCC and [25]. |
| Conclusions  Why are some key findings highlighted in the abstract but not reiterated or sufficiently addressed in the conclusion?  In abstract  These findings suggest that PBMAs offer a viable alternative for consumers in Malaysia seeking healthier and more sustainable food options, particularly for those aiming to reduce their intake of fat and sodium. However, PBMAs may not be recommended for individuals seeking a higher protein and lower carbohydrate diet. Further research is recommended to explore micronutrient profiles to enhance dietary decision-making.  In conclusion  Considering these findings, PBMAs present themselves as a viable choice for consumers in Malaysia in terms of availability, price, and nutritional composition (except carbohydrate). However, further research should include direct comparisons with local meat equivalents, encompassing both processed and unprocessed varieties, to better understand the complete nutritional benefits and limitations of PBMAs. Additionally, a thorough assessment of micronutrient content is essential to fully assess the health implications of these products, ensuring that consumers are well-informed about their dietary choices. | A sentence in Conclusion has been rewritten to better reflect the consistency with Abstract, as follows:    “Considering these findings, PBMAs present themselves as a viable choice for consumers in Malaysia who are concerned about healthier and more sustainable food options, in terms of availability, price, and nutritional composition (albeit with the caveat of lower protein and higher carbohydrate).” |
| **Reviewer #5** |  |
| 1. Please define Abbreviation in first appearance. The abbr. PBMA must be defined in both the abstract and at the beginning of the introduction. | The abbreviation for PBMAs has been now defined in Abstract and at the beginning of Introduction. |
| 2. The article does not mention with a compelling argument for why the quantitative analysis of PBMAs in Malaysia is necessary. The narrative of rising demand in Malaysia does not convincingly signify the demand just by correlating it with broad global market trends. This leads to the broader description of problem; and strengthening this aspect will better justify the study objectives. | The following sentence was added to better justify the objectives:  “With the rise of PBMAs in Malaysia, Malaysian consumers would be interested to know whether these products are available in their neighbourhood, and how affordable are they, and how nutritious are they by themselves or when compared with meat equivalents.” |
| 3.  a. The study excluded small and medium-sized grocery stores, potentially missing a segment of the market that may offer different PBMA varieties or pricing structures. How will the authors address that?  b. How would you justify that selection criteria of supermarkets and hypermarkets via Google searches and on-site visits did not introduce any biases in the selection of supermarkets and hypermarkets.  c. Please correct "Supermarkets and Hypermarkets in Subang" to reflect multiple location name in order to avoid confusion that there was only one google search for Subang. | a. We performed a preliminary eyeballing survey at all small and medium-sized grocery stores, supermarkets and hypermarkets, and found that small and medium-sized grocery stores, whether independent or chain-based, offered limited amounts (less than 3) of PBMAs.  b. In our opinion, Google searches are pretty comprehensive in listing out the supermarkets and hypermarkets at a specific area or locality. Most of the time, the “Place” results pointing to Google Map locations will return as the top search result, as shown in the picture below:    Sentence amended to “Searches such as "Supermarkets and Hypermarkets in Subang", "Supermarkets and Hypermarkets in Damansara" and henceforth, were performed…” |
| 4. In the methodology describing nutritional profiling, please explain Nutri-Score system. While the color scheme and scores are mentioned, please specify a detailed explanation for -15 (healthiest) to +40 (least healthy), similar to the caption of figure 4, for clarity and context. | Rationale and more information provided for Nutri-Score:  This scoring system was selected as it is a widely used front-of-pack labelling system that provides user-friendly information on the nutritional quality of food and beverages. This food-labelling system has been applied voluntarily in food products sold in European Union countries such as Sweden, Italy, and Germany; hence, nutrient profiling of PBMAs in these countries also utilised Nutri-Score [21–23]. |
| 5. Missing “Full stop” in line 204 and line 228. | Full stops were added. |
| 6. Line 280-292, supporting evidence for price comparisons between PBMA provides contradictory findings. Even though the PBMA prices in Canada are similar to those in Malaysia, the cost of meat in Canada is not significantly different than Canadian PBMA. This undermines the argument that PBMA are expensive. It is recommended to include additional references to further support the price comparison.  Furthermore, please discuss how the price variations across regions or product categories affects consumer behavior, if possible. | Paragraph in Discussion on price analysis and comparisons has been rewritten, as follows:  A study in Canada reported PBMA prices at USD 1.95 per 100g, with no significant price difference with their meat equivalents (USD 1.81 per 100g); however, the price of plant-based cheese and yogurt analogs was higher than their animal-based counterparts [9]. In 2022, a market survey found that plant-based beef alternatives were priced higher at approximately USD 6.70/pound (or USD 1.48/100g), while beef costs about USD 5/pound (or USD 1.10/100g) [32]. Also, New Food Innovation reported that plant-based meat remains 67% more expensive than animal meat in the UK [33]. Compounded by the ongoing cost of living crisis and rising inflation, this price parity poses a significant barrier for consumers looking to choose more sustainable alternatives, making it even harder for people to make the switch. Indeed, an unpublished thesis reported that among 227 Malaysian respondents, 44.4% stated expensive prices as one of the reasons for not purchasing PBMAs [17]. |
| 7. The PBMAs generally have lower energy densities due to factors like lower fat, higher water, fiber content, and hydrocolloid binders (replacement of fat that yields more energy) compared to meat products. However, the comparison between Malaysian PBMAs and European meat products (lines 296) is ambiguously presented. The phrase “Malaysian PBMAs generally have lower energy densities than European meat-based products” suggests that European PBMAs may have higher energy density, which could be misleading. ……In fact, rewriting this whole paragraph would do the justice to the manuscript (Rewrite line 294-308). | The whole paragraph has been rewritten, as follows:  When comparing plant-based meat alternatives (PBMAs) in Malaysia with those in Europe (Table S5), both have similar energy densities, meaning they offer comparable caloric content, with slight differences depending on the product category. However, Malaysian PBMAs generally have lower energy densities than European meat-based products, which could benefit consumers aiming to reduce their calorie intake. In terms of protein content, Malaysian PBMAs consistently contain less protein than both European PBMAs and meat-based products, highlighting an area for potential improvement. On the other hand, Malaysian PBMAs tend to have similar or lower total fat content compared to their European counterparts. However, they often contain higher levels of saturated fat. Compared to meat-based products, Malaysian PBMAs generally have lower total and saturated fat content, although some categories still require optimization. Malaysian PBMAs also typically contain more carbohydrates than European PBMAs and meat-based products, likely due to the use of additional fillers and binding agents, which may impact the product’s nutritional balance and glycaemic load. Additionally, Malaysian PBMAs tend to have higher sodium levels than European PBMAs, although their sodium content is generally lower than that of meat-based products, despite still being relatively high. |

We are hopeful that the above responses and amendments have satisfied the requirements of esteemed reviewers, and it is our great hope that our manuscript will be accepted for publication.

Yours sincerely,

Yee-How Say.
