# Supplementary material for: Availability, price and nutritional assessment of plant-based meat alternatives in hypermarkets and supermarkets in Petaling, the most populated district in Malaysia
Source: PLoS One. 2024 Dec 12;19(12):e0309507. doi: 10.1371/journal.pone.0309507 (PMC11637325; doi:10.1371/journal.pone.0309507)
Supplement: Attachment — Submitted filename: PLOSONE PBMA Response to Reviewers2.docx [file pone.0309507.s010.docx]

**PONE-D-24-34533R1**

**Availability, Price and Nutritional Assessment of Plant-Based Meat Alternatives in Hypermarkets and Supermarkets in Petaling, the Most Populated District in Malaysia**

**Katty Jia Qi Lou, Nadia Nantheni Rajaram, Yee-How Say**

**PLOS ONE**

Dear Academic Editor,

We thank you and the reviewers for the constructive comments and we have addressed them point-by-point and amended the manuscript, as follows:

| **Comments** | **Response to Reviewers** |
| --- | --- |
| **Reviewer #2** |  |
| 1. I suggest that the paper will be more insightful if you can highlight the narrative regarding the effect of processed or junk foods compared to semi-processed or whole-grain foods on health with supporting from existing literature. | The following narrative has been added to discussion:   - Highly processed foods and junk foods are typically calorie-dense but nutrient-poor. They are engineered for flavour, often using refined sugars, high-fructose corn syrup, unhealthy trans fats, and a host of preservatives [44]. The long-term consumption of these foods can lead to significant health problems, including obesity, CVD, diabetes, cancer, and poor mental health [44]. In contrast, semi-processed foods and whole grains - such as whole-wheat bread, brown rice, quinoa, oats, and minimally processed vegetables [45] - are rich in essential nutrients like fiber, vitamins, and minerals. These foods are digested more slowly, resulting in a steadier supply of energy and numerous health benefits like weight management, heart health, improved digestive health, lower risk of chronic diseases, and better mental health [46,47]. - Regardless of the classification of processing of PBMAs, we opine that it is important to have a balanced diet by complementing PBMAs with whole grains and unprocessed/semi-processed vegetables and fruits for even better health benefits. |
| 2. Again, the results presentation in the discussion section seems to be a duplication; rather, stick to the possible reasons and consequences, and comparison of the finding with previous works is important. | We have already avoided the duplication of results in the discussion section in our first revision. Nevertheless, we have further removed the repetition of results, as follows:   - “Compared to the median price per 100g of the least expensive PBMA category (Pieces/Chunks/Fillets/Strips at MYR 2.98 or ~USD 0.7), PBMAs are at least twice as expensive as chicken, which has a ceiling price of MYR 1.14 (~USD 0.3) per 100g [31]. Categories such as Burger/Patties and Minced Meat were the most expensive, possibly due to a high percentage of imported products” changed to: “The ceiling price of poultry in Malaysia is MYR 1.14 (~USD 0.3) per 100g [31] and therefore, PBMAs are at least twice more expensive compared to the median price of the least costly category - Pieces/Chunks/Fillets/Strips. Categories such as Burger/Patties and Minced Meat were the most expensive, possibly due to a high percentage of imported products.” - “Our data also showed that most PBMA categories had lower total fat and saturated fat content compared to their meat equivalents. The fats in PBMAs primarily come from vegetable oils, such as soybean, sunflower, and olive oils, which contain polyunsaturated and monounsaturated fats, unlike the saturated fats found in greater amounts in meat [5]” rewritten to “The lower total fat and saturated fat contents of PBMA categories compared to their meat equivalents is due to ingredients of the former primarily coming from vegetable oils like soybean, sunflower, and olive oils containing polyunsaturated and monounsaturated fats [5].” - “Most PBMA categories in this study had lower sodium content than their meat equivalents, except for Pieces/Chunks/Fillets/Strips, which had significantly higher sodium levels. This contradicts other studies that consistently found higher sodium content in PBMAs” rewritten to “Except for Pieces/Chunks/Fillets/Strips, the Malaysian PBMAs surveyed in this study had lower sodium content than their meat equivalents, contradicting with previous studies in other countries which found higher sodium content in the former”. - “Our results showed PBMA categories ranged from Grade A to Grade C, with more than half falling under Grade C and Nutri-Scores ranging from 3 to 10. Prepacked Cooked Meals had the highest mean Nutri-Score of 7.88, likely due to their higher energy density and sodium content from added oils and sauces: rewritten to “The Malaysian PBMAs surveyed in this study had Nutri-Score ranging from Grade A to Grade C, with Prepacked Cooked Meals having the highest mean, likely due to their higher energy density and sodium content from added oils and sauces.” |

We are hopeful that the above responses and amendments have satisfied the requirements of esteemed reviewers, and it is our great hope that our manuscript will be accepted for publication.

Yours sincerely,

Yee-How Say.
